# Measuring pain and nociception: Through the glasses of a computational scientist. Transdisciplinary overview of methods

**DOI:** 10.3389/fnetp.2023.1099282

**Published:** 2023-02-10

**Authors:** Ekaterina Kutafina, Susanne Becker, Barbara Namer

**Affiliations:** ^1^ Institute of Medical Informatics, Medical Faculty, RWTH Aachen University, Aachen, Germany; ^2^ Faculty of Applied Mathematics, AGH University of Science and Technology, Krakow, Poland; ^3^ Clinical Psychology, Department of Experimental Psychology, Heinrich Heine University, Düsseldorf, Germany; ^4^ Integrative Spinal Research, Department of Chiropractic Medicine, University Hospital Balgrist, University of Zurich, Zurich, Switzerland; ^5^ Department of Cognitive and Clinical Neuroscience, Central Institute of Mental Health, Medical Faculty Mannheim, Heidelberg University, Mannheim, Germany; ^6^ Junior Research Group Neuroscience, Interdisciplinary Center for Clinical Research Within the Faculty of Medicine, RWTH Aachen University, Aachen, Germany; ^7^ Institute of Physiology, Medical Faculty, RWTH Aachen University, Aachen, Germany

**Keywords:** computational models, interdisciplinary communication, measurements, pain, nociception, transdisciplinary research

## Abstract

In a healthy state, pain plays an important role in natural biofeedback loops and helps to detect and prevent potentially harmful stimuli and situations. However, pain can become chronic and as such a pathological condition, losing its informative and adaptive function. Efficient pain treatment remains a largely unmet clinical need. One promising route to improve the characterization of pain, and with that the potential for more effective pain therapies, is the integration of different data modalities through cutting edge computational methods. Using these methods, multiscale, complex, and network models of pain signaling can be created and utilized for the benefit of patients. Such models require collaborative work of experts from different research domains such as medicine, biology, physiology, psychology as well as mathematics and data science. Efficient work of collaborative teams requires developing of a common language and common level of understanding as a prerequisite. One of ways to meet this need is to provide easy to comprehend overviews of certain topics within the pain research domain. Here, we propose such an overview on the topic of pain assessment in humans for computational researchers. Quantifications related to pain are necessary for building computational models. However, as defined by the International Association of the Study of Pain (IASP), pain is a sensory and emotional experience and thus, it cannot be measured and quantified objectively. This results in a need for clear distinctions between nociception, pain and correlates of pain. Therefore, here we review methods to assess pain as a percept and nociception as a biological basis for this percept in humans, with the goal of creating a roadmap of modelling options.

## 1 Introduction

Pain is a complex phenomenon with enormous impact on quality of life and everyday life functions. The International Association for the Study of Pain (IASP) currently defines pain as following (Raja et al., 2020): “An unpleasant sensory and emotional experience associated with, or resembling that associated with, actual or potential tissue damage.” Acute pain mechanisms in healthy states are the result of evolutionary development (Bonavita and De Simone 2011) that fulfil essential functions in information feedback loops which are essential for survival (Armstrong and Herr 2022). Conversely, clinical pain, for example chronic cancer pain (Bennett et al., 2019), painful neuropathies (Marchettini et al., 2006), as well as chronic primary pain (Treede et al., 2015) might at the beginning signal a change of wellbeing possibly leading to recreational behavior or avoidance, but provides limited information within the acute feedback loop and strongly affects main aspects of life and wellbeing.

The development of computational methods in the last decades allowed for construction of computational models that promote a better understanding of pain mechanisms [see e.g., the recent systematic review of Lang et al. (2021)]. Those models span from single neuron levels (Chrysostomidou et al., 2021; Le Franc and Le Masson 2010; Balachandar and Prescott 2018; Tigerholm et al., 2014) over spinal synaptic effects (Tanaka et al., 2021) to brain imaging analysis (Morton et al., 2016). However, the complexity of nociceptive signaling and pain perception calls for further development, building multiscale and integrative models (Crodelle et al., 2019; Ionescu et al., 2019; Mendell 2011; Kucyi and Davis 2017; Seymour and Mancini 2020; Medlock et al., 2022) to generate more comprehensive insights. Thiam and colleagues, (Thiam et al., 2019), proposed deep physiological models, which combine deep learning and multi-modal data fusion to automatic pain detection. The work by Akal et al. (2022) shows how machine learning can make use of clinical information to support diagnostic decisions. As a matter of fact, the relatively recently defined field of network physiology (Bashan et al., 2012) provides even wider perspectives on the modelling possibilities, with holistic view of different aspects of human physiology. Within this framework, pain can be seen not only as a complex physiological phenomenon with many interacting components, but also as a manifestation of broader picture of abnormal excitability properties in neural system which can also show through e.g., epileptic seizures.

To ensure the successful development of such comprehensive models, it is necessary to build interdisciplinary collaborative teams and facilitate the communication between partners with medical, biological, or psychological backgrounds on one hand and with computational backgrounds on the other. Such communication can be supported by reviewing articles on certain topics, because they give a bird’s eye overview and keep the usage of domain-specific terminology to a minimum. See for instance Dubin and Patapoutian (2010) and Middleton et al. (2021) for reviews on nociceptors, Julius and Basbaum (2001) for information on molecular mechanisms of nociception and Yam et al. (2018) for pain neurotransmitters review.

Computational models largely rely on experimental measurements and assessments. Therefore, it is essential to understand the available choice of such measurements and assessments, their potential and limitations. Of uttermost importance in this context is the fact that nociception does not equal pain perception. Nociception is, as defined by IASP, “the neural process of encoding noxious stimuli.” In contrast, pain is, by definition, a subjective phenomenon. Many factors, including stimulus characteristics, biological aspects as well as psychological and social influences modulate how we perceive pain. Such modulatory influences impede the assessment of pain perception, hindering objective measurements. Thus, we rely largely on subjective assessments such as self-reports in experimental and clinical contexts.

There are several closely related overviews available that are well-suited for non-expert readers. In particular, the paper of Johnson (2016) focuses on pain-related measurements in animal research and Deuis et al. (2017) on pain-related behavior in rodents. There is a large overlap between the methods used for animals and humans, however for many reasons such as ethical concerns, communication, physiological differences etc., we find it important to separately review the state of human research.

Some important approaches to assessing human pain perception and nociception are based on measuring brain responses to stimuli with electroencephalography (EEG) and functional magnetic resonance imaging fMRI [see (Kramer et al., 2012) and (Xu and Huang 2020) for short overviews]. Recent developments of wearable sensors provide indirect indicators of pain *via* physiological stress responses [see e.g., (Chen et al., 2021)].

Another facet of quantitative pain assessment is represented by research on biomarkers of pain. The review paper of Tracey et al. (2019) provides a broad overview of the topic and the paper of Niculescu et al. (2019) focuses on precision medicine based on blood biomarkers of pain. The second study used blood gene expression biomarkers that were predictive of pain state, and of future emergency department visits for pain in psychiatric patients and found MFAP3 (a component of elastin-associated microfibrils) significantly correlating.

All these reviews focus on different subtopics of measuring pain. This manuscript rather aims to provide a basic overview of pain-related assessments in humans that are of interest in the context of computational models of nociception and pain. Some references to animal research are given to fill existing gaps in human-centered models. The review does not aim to give an exhaustive list of methods, but rather a roadmap of the research domain. The large variety of available approaches is summarized, and special attention is paid to advantages and limitations of these approaches in assessing the experience of pain. The manuscript is specifically targeted to data scientists and computational experts, interested in applications in the domain of pain research.

**FIGURE 1:**
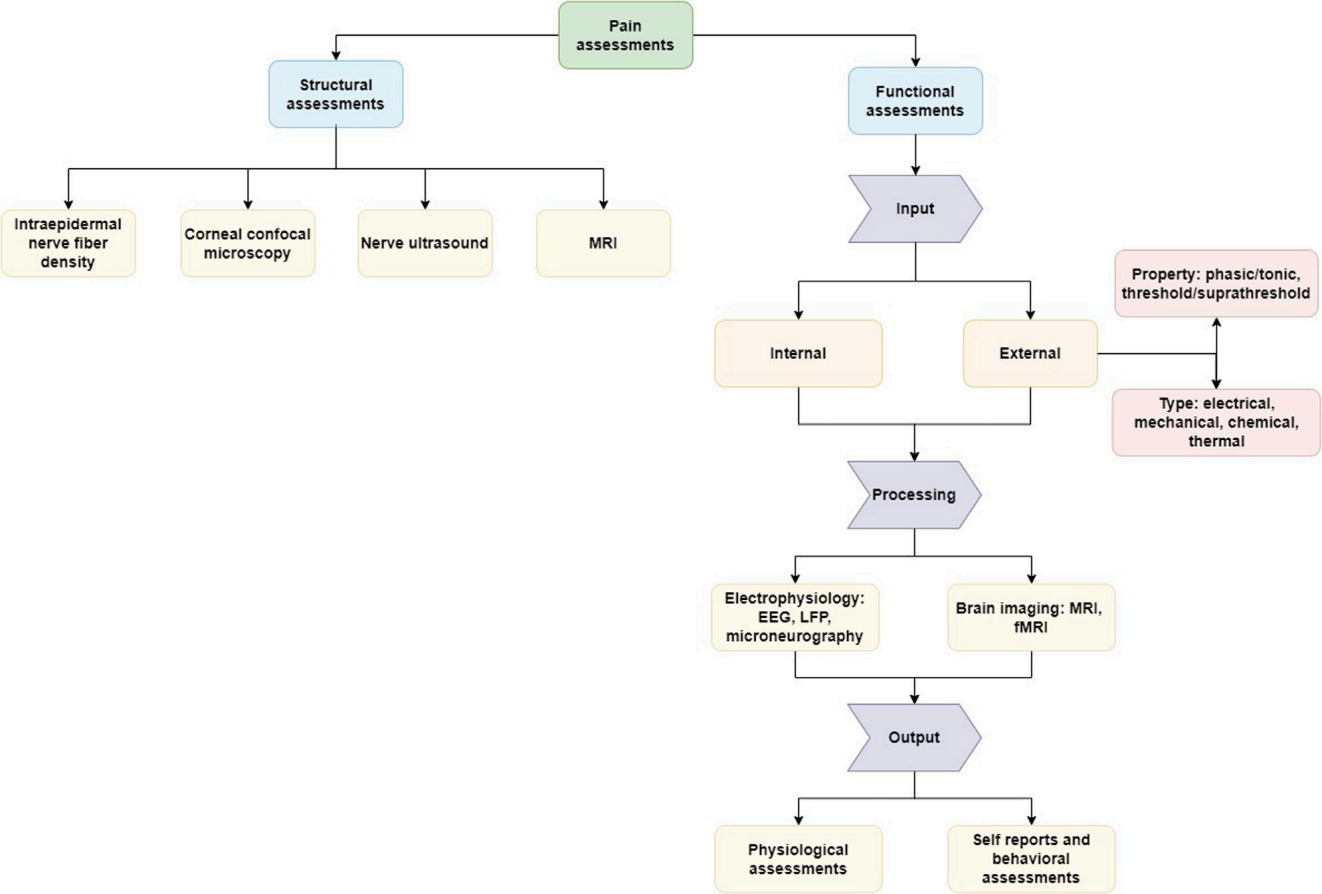
Representation of the different aspects of the assessment of pain and nociception. Structural assessments focus on morphological features (e.g., tissue property) and functional assessments on the process of signaling between the external stimulation or internal pathological trigger and the experience of pain. Illustrated are assessment methods reviewed here as examples of common approaches and tools without the aim of providing a comprehensive overview of all methods available.

For this purpose, we organize the described methods as presented in Figure 1. The first category, i.e., structural or morphological assessments, includes any examinations related to tissue properties at a given time point. In contrast, the second category, i.e., functional assessments, can be seen as an insight into the information processing, because the assessed data is linked to the input, induced for example by stimulation techniques. The translation of this input to measurable output can be seen as the route from nociception to pain which can be modelled as a computational process. Structural assessments may be repeated in time to investigate the relationship between the anatomical changes and nociceptive processes.

The section on structural assessments has no further sub-sections and describes the reviewed methods directly, while the part about functional measurements is further subdivided based on the relevant part of the signaling process (input, processing, output). The idea of a computational process from external input *via* signal processing to the experience of pain as output can be applied well in healthy states. However, external input does not necessarily need to be present, because the perception of pain can be also generated by endogenous input (e.g., induced by movement, inflammation, etc.) and by disturbances or pathologies in the signaling process, in particular in disease states such as chronic pain (Djouhri et al., 2006; Samineni et al., 2017). Along with this logic we will follow in the review different methods to assess nociception, the underlying “hardware” in morphological aspects and the perception of pain.

## 2 Structural assessments

Common assessments in the context of pain include structural or morphological assessments of relevant physiological underpinnings at different levels of the neuraxis, from peripheral to central aspects. Particularly, in clinical contexts, structural assessments are a common tool, for example, to study the integrity of nociceptive pathways. Thus, structural assessments in the context of pain research are focused typically on quantification of a given part of the nervous system, for example, density assessments of peripheral nerve fibers or brain grey matter thickness. Those measures are linked to different parts of the pain signaling system and have different levels of accuracy, but what is very important to consider, is that outcomes of such structural assessments are in most instances not directly related to perceived experimental and clinical pain. While alterations found in such structural assessments, for example in chronic pain, can give information on changes in the nervous system in disease states, these changes cannot be considered as objective markers of pain. Because of their importance, in particular in medical contexts, we include a brief overview of the most common assessments here. Specifically, we want to emphasize in this context that such assessments cannot be equated with the subjective experience of pain.

### 2.1 Intraepidermal nerve fiber density (IENFD)

Nociceptors (McArthur et al., 1998) are the neurons that bring information from peripheral tissues to the central nervous system *via* electrical signals (“action potentials”) traveling along their axons to the spinal cord. Thus, it appears obvious to assess structural changes of such nociceptors, particularly in patients with pain, because of suspected damage of the nerve fibers as an assessment of the underlying peripheral “hardware” of pain. The easiest way to access such nociceptors in humans is a skin biopsy and immunohistochemical staining of the nociceptors in the skin. To characterize the nerve fiber density, peripheral nociceptive nerve fibers can be stained in skin biopsies using the marker anti–protein gene product 9.5 (PGP9.5) (McArthur et al., 1998; Ebenezer et al., 2007; Lauria et al., 2010). Density of the nerve fibers is quantified by counting the number of nerve fibers crossing the barrier between epidermis and dermis in a skin sample using a microscope. Attempts have been made to differentiate between the number of nerve fibers in the epidermis and the layer below, i.e., the dermis, staining for neuropeptides (chemical messenger synthesized in neurons) or describing the morphology such as axonal swellings and number of branches (Karlsson et al., 2015; Cheung et al., 2015; Schley et al., 2012; Kalliomäki et al., 2011; Karlsson et al., 2021). While a loss of skin innervation correlates with decreased sensitivity to acutely evoked pain stimuli, for example in terms of heat pain threshold, typically no such correlation can be found with ongoing clinical pain. Nevertheless, some evidence suggests a correlation between deeper lying dermal fibers containing neuropeptides and ongoing pain in diabetic neuropathy (Karlsson et al., 2021). A model of the axonal tree in the skin revealed that more branches can lead to signal amplification, suggesting a link between morphology and function (Barkai et al., 2020). However, another problem in correlating pain with intraepidermal nerve fiber density is that there are no morphological differences between non-nociceptive thermo- or mechanoreceptors and nociceptive fibers. Considering the above, at the current state intraepidermal nerve fiber density cannot be used as a correlate of human pain perception.

### 2.2 Corneal confocal microscopy

To assess specifically small nerve fiber characteristics in the cornea, corneal confocal microscopy (CCM) can be used, as a rapid non-invasive ophthalmic imaging technique. Similar to nerve fiber density in skin, this technique assesses corneal nerve fiber density. In addition, branch density, fiber length, and inferior whorl length can be assessed (Lukashenko et al., 2021; Petropoulos et al., 2021). Compared to skin biopsies, CCM has the advantage that it is non-invasive. Similar to the measure of the number of intraepidermal nerve fibers (IENFD) this method is useful to assess morphological changes of nociceptors such as small fiber neuropathy or diabetic neuropathy (Cosmo et al., 2022) and changes in nociceptors in other degenerative diseases such as Parkinson’s disease (Kass-Iliyya et al., 2015). Nevertheless, the relation of these measures to pain perception is not clear, similar to nerve fiber morphology in skin biopsies. Additionally, it is not clear if and how findings in the cornea can be generalized to the rest of the body.

### 2.3 Nerve ultrasound

Nowadays it is possible to use high resolution ultrasound to show single fascicles in a peripheral nerve. Therefore, this method can be used to examine structural changes such as the alteration of nerve fascicles diameter. Similarly to intraepidermal nerve fiber density or corneal nerve fiber structure, nerve ultrasound is useful in assessing damage of so called “thin nerve fibers” including nociceptors. It is currently used to detect morphological changes in neuropathies (Cosmo et al., 2022) and nerve entrapment syndromes such as carpal tunnel syndrome (Lin et al., 2022). However, neither a direct link to evoked pain nor to ongoing pain has been shown so far.

### 2.4 Structural magnetic resonance imaging of the brain

Based on magnetic resonance imaging (MRI), structural characteristics of the human brain can be described and some of them have been related to pain (Baliki et al., 2011; Kregel et al., 2015; Kumbhare et al., 2017). In particular, changes in such characteristics have been described in patients suffering from chronic pain and a pathogenetic relevance has been discussed [e.g., (Zhang et al., 2019a; Martucci and Mackey 2018)]. One common measure in this context is the thickness and/or volume of brain grey matter in different brain regions, which can be estimated using standard MRI analyses techniques. Most commonly, reduced thickness of grey matter in certain brain regions, such as frontal areas, anterior cingulate cortex, and the insula, has been described in chronic pain compared to healthy controls (Ma et al., 2022; Wang et al., 2022). However, the exact relationship of grey matter thickness/volume and the perception of pain and the development and maintenance of chronic pain remains unclear.

Another common structural assessment based on MRI is diffusion tensor imaging (DTI). Based on indirect measurements of the degree of anisotropy and structural orientation, tracks of fiber in the white matter are estimated (Kumbhare et al., 2017; Rahimi et al., 2022). DTI is used to study the architecture of white brain matter in health and disease states (Soares et al., 2013). Alterations in estimated fiber tracks in disease states compared to healthy controls are interpreted as pathogenetic changes in brain structure. DTI is commonly used in human pain research, but as with the estimates of grey matter thickness, no direct link to pain perception and the development of chronic pain exists.

In sum, structural assessments of the human brain have gained interesting insights by showing alterations in structural brain characteristics in chronic pain, but they do not allow specific conclusions on the perception of experimental or ongoing/clinical pain (Davis and Seminowicz 2017). Overall, it is not surprising that no or only minor correlations can be found between clinical pain and structural brain correlates of pain, because these structures and their potential changes are not specific to chronic pain. Similar changes in the same structures can be also observed in different clinical conditions.

## 3 Functional assessments

Functional assessments of pain in humans cover a much broader range of options than structural assessments. In addition, these functional assessments are typically more closely related to the experience of pain. Thus, the output that is assessed is typically related to (external or internal) input, with this relation being defined by the signaling processes in between. However, input and output are not necessarily related linearly or in a 1:1 fashion. The exact relationship is influenced by many modulating factors and shows large inter- and intra-individual variations. This is one of the main points where computational modelling and data-driven network models come into play, offering new and promising routes to gain insights in how the subjective experience of pain is created. A fundamental basis for such models is an understanding of how external input can be created and controlled, available indicators of the signaling process, and, finally, which output measures can be used and what these measures indicate.

### 3.1 External input

External input to evoke painful sensations can be induced in several modalities, such as mechanical and thermal stimuli that target at least partially different nociceptors, for example mechanosensitive (responsive to mechanical stimuli) and mechano-insensitive (silent, sleeping, class 1b, not responsive to mechanical stimuli under physiological conditions) nociceptors [see (Dubin and Patapoutian 2010) and (Middleton et al., 2021) for overview on nociceptors]. Other features of nociceptive stimuli, some of them being inherent to the stimulation technique and modality, are functionally relevant with respect to the induced nociceptive signaling and the resulting pain experience. For example, the duration of the stimulation plays an important role. Phasic stimuli are short-lasting whereas tonic stimuli last longer and the duration itself shapes the sensation tremendously. Further, the area of stimulation varies and with this the pain sensation as well. For example, while mechanical stimulation using pin prick (see Section 3.1.2.2) affects only a very small point on the skin, thermal stimulation affects typically at least an area of 1 cm^2^ and thus many more nociceptors in the skin. As a result, different levels of spatial and temporal summation are caused by different stimuli. Moreover, external input can be applied with different intensities thereby strongly modulating the strength of the perceived pain, for example allowing to describe individual “dose-response curves.” While these differences might appear subtle, they are important to be considered in pain research, because choosing non-fitting input leads to useless information. Similarly, each stimulation technique has to be applied correctly to result in interpretable data. For example, skin properties and preparation can strongly influence resistance and thus how applied electrical stimuli are felt. Different levels skin types are innervated differentially affecting the resulting perception from thermal stimulation. Since stimulation protocols can vary largely depending on the specific application and the research question, we cannot detail all these protocols here.

#### 3.1.1 General aspects of nociceptive stimulation

##### 3.1.1.1 Phasic versus tonic stimulation

Phasic stimulation mimics acute painful stimulation and is linked to a feedback mechanism necessary to remove the cause of the painful sensation. In contrast, tonic stimulation is used to resemble states of injury which prioritize recovery (Zhang et al., 2018). Phasic and tonic stimulation can be used in experimental settings to assess different functions of the pain system. Specifically, tonic stimulation, for example using the Capsaicin-Heat Model (Price et al., 2018; Meeker et al., 2022) allows painful stimulation of more than 1 h duration and can be used to assess functions that are also relevant in clinical settings. Related to the length of the stimulation as well as the frequency of repeated stimuli, temporal summation can occur. It influences the threshold by synaptic effects (Tanaka et al., 2021) and manifests in an increase in the pain experience. It can also strongly modulate the perception of pain. Depending on the stimulus intensity not only temporal summation and thus sensitization [the effect of increased reactivity to the same stimulus (Latremoliere and Woolf 2009)] can occur, also the opposite process is possible, namely habituation. It seems that habituation tends to occur with stimulation intensities close to the individual pain threshold and temporal summation/sensitization with higher intensities (Kleinböhl et al., 1999). Similarly, the stimulus modality seems to determine whether habituation or sensitization is more likely to occur. For example, electrical stimulation commonly induces strong habituation. Such effects of temporal summation/sensitization and habituation need to be taken into account with experiment pain stimulation because they strongly affect the pain experiences resulting in dynamic changes of the course of stimulation and/or testing.

##### 3.1.1.2 Threshold versus supra-threshold testing

In psychophysical testing two different methods typically are used: assessing the discrimination and pain threshold or using suprathreshold stimuli that are evaluated by participants, for example, in terms of perceived intensity and un/pleasantness. Describing in detail different methods for those two approaches is beyond the scope of this short review. It should be noted, however, that threshold testing assesses most likely other mechanisms than suprathreshold, which can be also used to test for different signal coding abilities of peripheral nerve fibers such as maximal discharge frequencies, adaptation, or recruitment of different peripheral nerve fiber types. For example, phasic mechanical stimuli activate mechano-sensitive C-fibers (polymodal nociceptors, class 1a fibers) in humans, but not mechano-insensitive C-fibers (sleeping nociceptors, mechano-insensitive nociceptors, sleeping nociceptors or class 1b fibers). Those sleeping nociceptors are under physiological conditions insensitive to mechanical stimuli, but can be “awaken” by sensitization: tonic pressure or pinching activates mechano-insensitive nociceptors after 1 min pressure, while mechano-sensitive nociceptors stop discharging signals after 1 min of pressure application. In parallel to differential processes, pain ratings typically increase over 2 min of pressure. Mechano-insensitive nociceptors are predominantly chemo-heat nociceptors and involved in chronic pain states such as inflammatory and neuropathic pain (Schmidt et al., 2000).

Further, suprathreshold testing involves also different central mechanisms than threshold testing. With threshold testing, the stimulation is terminated when the threshold is reached and thus at low discharge frequencies of nociceptors. Correspondingly, temporal summation is not involved or only to a minor extent in threshold testing. However, such temporal summation is known to be relevant in states of ongoing pain. For example, 1 min sine wave stimulation causes pain in both healthy individuals and neuropathic pain patients in the beginning. However, at the end of the stimulation, those two groups show differential responses with healthy individuals showing decreases and pain patients showing increases in their pain ratings. Suprathreshold testing involves specific central mechanisms such as modulation of synaptic transmission in the spinal cord, which are modulated by longer trains with higher frequency of signals arriving at the synapse, which is more relevant for long term states with ongoing pain.

#### 3.1.2 Stimulation modalities

Modalities that are commonly used to generate external input in pain research are electrical, mechanical, chemical, and thermal stimulation. These modalities result in different subjective experiences and are used for different purposes. The aim of these descriptions is to give a brief overview of commonly used stimulation techniques in human pain research. Accordingly, we give here a very short overview focusing only on some specific aspects, which are inherent to the nature of each modality, and which should be considered. All these stimulation techniques fulfil the purpose to induce different types of pain to assess responses to these stimuli on very different levels (e.g., autonomic, motor/behavioral, verbal responses), which will be addressed in the “output” subsection.

##### 3.1.2.1 Electrical stimulation

Electrical stimulation is typically applied using constant current stimulators and cutaneous or intra-dermal electrodes. Perceived pain varies depending on electrode configuration, resulting in different current densities, and on the specific pulse form, frequency, and intensity. With high enough current densities electrical stimulation activates all nerve fibers in a given skin area independent of their receptive properties. Importantly, specific pulse forms in combination with different electrode configurations (Poulsen et al., 2022) can be used to differentially activate fiber subclasses, such as fast conducting A-beta fibers, medium conducting A-delta fibers or slowly conducting C-fibers. Moreover, even within C-fibers different subclasses can be targeted. Short phasic stimuli activate A-delta fibers, while longer pulses especially with a sine wave form activate preferentially C-fibers (Jonas et al., 2018; Rukwied et al., 2020). Electrodes with a small surface lead to high current densities and are more likely to activate C-fibers than electrodes with a large surface. Special configurations of anode and cathode, e.g., concentric electrodes with an intraepidermal part, seem to preferentially activate A-delta fibers mediating pain and thermal stimuli compared to A-beta fibers, which mediate non-painful sensations (Poulsen et al., 2020; Poulsen et al., 2021). High frequency stimulation activates preferentially A-fibers, while C-fibers cannot follow higher stimulation frequencies over 100 Hz for longer time periods. Particularly, sleeping nociceptors, which are thought to play an important role in inflammatory and neuropathic pain, can follow only very low frequencies for longer time periods [<50 Hz, (Werland et al., 2021)]. Activating this special class of sleeping nociceptors with electrical stimulation is challenging, because they have high thresholds for rectangular pulses. Using sine wave stimulation allows to activate sleeping nociceptors, but this does not activate A-fibers (Jonas et al., 2018). So far, the only electrical stimulation paradigm that correlates with ongoing pain in patients with neuropathic pain is a lack of adaptation to sine wave stimulation of 1 min duration with 4 Hz (Jonas et al., 2018). The optimal stimulus to activate so called mechanosensitive C-fibers, which are thought to transmit the discriminative aspects of acute nociceptive and painful stimuli, is a slowly depolarizing ramp such as a 500 ms long half sine wave (Rukwied et al., 2020; Tigerholm et al., 2020). Electrical stimulation has also been used to induce hyperalgesia (increased pain sensation to a slightly painful stimulus), allodynia (painful sensation upon non-painful stimulation), and central sensitization (sensitization in central nervous system) as human surrogate models of hyperalgesia (Koppert et al., 2001; Vo and Drummond 2013; Klein et al., 2004). Electrical stimulation is also used as a negative modulator of pain in the form of pain inhibition (Gibson et al., 2019; Klein et al., 2004; Fogel and Winfree 2022) e.g., well known as transcutaneous electrical nerve stimulation (TENS) for which machines can now be bought in drugstores. Both phenomena could be explained by long-term potentiation or depression respectively, which are mechanisms of the central nervous system that lead increasing and decreases responses to succeeding stimuli relative to the first stimulus. To give an extensive overview over the field of electrical stimulation as modulator of pain is beyond the scope of this review and can be found, at least in part, in the review of human surrogate models of central sensitization of Quesada and colleagues (Quesada et al., 2021).

##### 3.1.2.2 Mechanical stimulation

Different techniques can be used for mechanical stimulation with a major difference between these techniques being the size of stimulation area. While so called “von Frey filaments” and “pin pricks” stimulate only very small skin area, pressure algometers and impact stimulators typically have stimulation radius of 1 cm or more. Von Frey filaments are constructed as a handle and a column of thin nylon fibers. The columns differ in their physical properties so that different mechanical force is necessary for their buckling. Similarly, pin pricks are tactile stimulators with a flat contact area of 0.25 mm in diameter for distinct different pressure intensities (between 8 and 512 mN). The skin area tested with von Frey hairs or other point like stimulators is usually very small containing often only 1 mm^2^. This leads only to spurious spatial summation in contrast to thermal stimuli, which are typically applied on an area of several cm^2^. Thus, a loss of epidermal innervation might cause bigger effects when using this type of stimulation and it is hard to produce a relevant pain sensation under physiological conditions without skin damage.

In contrast, pressure algometers (mechanical or electronical) allow continuous application of increasing pressure. However, such algometers do not only stimulate the skin, but also deeper structures such as muscle and periosteum, which is very sensitive to pain. Similarly, impact stimulation affects also deeper structures, by which another component of deep pain in contrast to pure skin pain is added and another level of spatial summation is reached.

All those stimuli target the skin or skeletal muscles, seldomly bones and periosteum, but not the viscera. Much less is known about visceral pain because of the bad accessibility, especially in human, for pain models and electrophysiological investigations. Often, mechanical stimulation is used for causing visceral pain. Extending balloons which can induce pressure pain, e.g., in the rectum and esophagus (Nozu and Kudaira 2009), are used to cause tonic stimulation.

Mechanical allodynia (see Section 3.1.2.2) can be tested on the skin by striking the skin lightly with a brush as described in the quantitative sensory test battery of the German network for neuropathic pain (Rolke et al., 2006).

##### 3.1.2.3 Thermal stimulation

Thermal stimulation is most commonly applied using computer-controlled contact thermodes for heat and cold stimulation on the skin surface (Zaric et al., 1996; Nguyen et al., 2004; Lithfous et al., 2020; Lithfous et al., 2022). Thermal stimuli are used varying broadly in intensity, duration and/or slope of rise and fall times and allowing for testing different components of pain perception, phasic or tonic pain, different peripheral nerve fiber types, and central signal processing mechanisms. Compared to thermodes, radiant heat has the advantage that it avoids simultaneous activation of touch and pressure sensitive nerve fibers in the skin. However, radiant heat is to a much lesser extent used due to a lack of certified human stimulators. Another type of thermal stimulation is laser stimulation (Plaghki and Mouraux 2005). Mainly in combination with measuring evoked potentials, so called laser evoked potentials (LEP), laser stimulation is commonly used for phasic stimulation activating preferentially thin A-delta nerve fibers. Thermal stimuli usually affect a larger area and thus cause pain *via* spatial summation and are better suitable for inducing substantial tonic pain than mechanical stimuli. However, it has to be taken into account that sensitizing and desensitizing (response to a stimulus is reduced or lacking after strong activation) effects can occur with repetitive application of thermal stimuli.

Related to thermal stimulation, another technique is the cold pressor test (Lamotte et al., 2021). With this test, participants immerse their hand or foot in water typically of 0°C–8°C, inducing an intense pain sensation. Most often, the time how long participants can keep their hand/foot in the water is measured as the tolerance time. Because of its intensity, the cold pressor test is also used as a cardiovascular test.

Thermal stimulation in both modalities, heat and cold, is also used as a so-called conditioning stimulus in conditioned pain modulation tests. Conditioned pain modulation assesses a central mechanism of endogenous pain inhibition *via* a descending pain modulation system [mechanisms which origin in the brain and act on spinal cord to reduce nociceptive input, (Kennedy et al., 2016; Nir and Yarnitsky, 2015)]. A tonic painful stimulus is used to induce this inhibition which is then tested by a phasic stimulus at another body site. Reduced responsiveness in this test has been reported in some patients with chronic pain and suggests impaired capability of endogenous pain modulation in these patients (Petersen et al., 2019; Martel et al., 2019).

##### 3.1.2.4 Chemical stimulation

Chemical stimulation differs from mechanical, thermal and electrical stimuli, because when applied it cannot be stopped. Such a stimulation technique is, for example, a single capsaicin (i.e., the active ingredient of chili peppers) injection that causes intense pain for a few minutes (Hughes et al., 2002; Scanlon et al., 2006; Balabathula et al., 2014). Thus, chemical stimulation is typically a tonic, suprathreshold stimulus. Chemical pain is often linked to inflammatory processes defined by pain, reddening and swelling. As such, capsaicin can be applied epicutaneously or intradermally to induce burning sensations or, if applied very focally, using the natural micro syringes of cowhage spiculea, the hairs on the seed pod of a tropical bean. Recently, continuous intracutaneous infusion of substances, e.g., solutions with low pH-value, have been demonstrated that they can be used to induce a well controllable chemical stimulus to evoke pain (Schwarz et al., 2017).

Many chemical substances are also used for sensitization, e.g., capsaicin causing heat hyperalgesia or menthol causing cold allodynia/hyperalgesia (Samuelsson et al., 2011). Capsaicin is also often combined with heat stimulation [Capsaicin-Heat Model (Price et al., 2018; Meeker et al., 2022)] to enable longer duration of stimulation without the risk of skin injuries and/or to enable the induction of potent pain relief perception. Even for a model of visceral pain chemical stimulation could be used (Hammer and Vogelsang 2007) by applying capsaicin activating the receptor TRPV1, *via* an endoscope in the duodenum or jejunum. Taken together, chemical stimulation is a tonic suprathreshold stimulus and therefore useful for modeling ongoing pain but hard to control and standardize in contrast to electrical, mechanical, and thermal stimulation.

### 3.2 Internal input

In many disease states, not stimuli coming from outside of the human body cause pain, but rather nociceptive processes from inside the body. Internal changes of the chemical milieu or mechanical stimuli from smooth muscle cells such as the intestines can cause activation of nociceptors and thus pain. Contractions of smooth muscle cells can cause strong pain such as during renal or biliar colics. Examples for endogenous chemical stimuli are a drop in pH and release of inflammatory mediators such as prostaglandin E2 (Dray, 1995; Rajamäki et al., 2013). Other substances deriving from metabolism can accumulate, for example, methylglyoxal in diabetes mellitus, which activates nociceptors *via* opening of ion channels (Eberhardt et al., 2012; Bierhaus et al., 2012). Further, infections can cause muscle or join pain, and headache either *via* the release of noxious substances by pathogens (e.g., lipopolysaccharides) or by immune reactions to pathogens such as release of interferons. Alternatively, the nerve fiber itself can initiate signal discharges as observed in some neuropathic conditions. In some conditions with nerve damage, for example in neuropathies, nociceptors seem to discharge “spontaneously” and send nociceptive signals to the spinal cord (Kleggetveit et al., 2012). However, in most conditions it remains unclear, if such discharges are truly spontaneous activity arising from the neurons themselves or if they are caused by chemical mediators such as methylglyoxal (Bennett, 2012). It is further unclear if differences between signal patterns of peripheral nociceptors exist between evoked and spontaneous activity. There might be also “spontaneous activity” along all centers of the nociceptive axis. For instance, damage to the thalamus can cause intense pain states (Dydyk and Munakomi, 2022). All those pain states in which an “internal cause” is present share the characteristics of suprathreshold and tonic pain.

### 3.3 Processing of nociceptive signaling

In the preceding section, we provided information on input that activates the nociceptive system. This information is conducted and processed at all stages of the nociceptive system from peripheral nerve endings to the spinal cord and the brain. At all these levels, information about signal processing can be gained by electrophysiological and imaging methods. In humans, available methods are restricted for ethical reasons and often indirect methods have to be used, because, for example, direct access to spinal neurons *via* recording electrodes on a single neuron level is not possible.

#### 3.3.1 Electrophysiology

Due to the nature of the neural system in which information is propagated *via* electrical currents, electrophysiological techniques can be considered core assessment tools for observation of signal processing related to nociception (Lefaucheur, 2019). One important and beneficial feature of electrophysiological methods is their very high time resolution. For instance, typical electroencephalography (EEG) devices used in clinical setups have a sampling rate of 256–1,024 Hz; for methods aiming at capturing individual action potentials, 20 kHz and more is a common standard. The available technologies cover a large variety of different spatial scales: from intracellular recordings to large networks of brain activity. The combination of those qualities makes electrophysiological methods very powerful tools for investigating the nervous system in general and nociceptive and pain signaling in particular. As this goes beyond the scope of the present review, the principles of electrophysiology are summarized in the paper of Narahashi (2003) and a further review of different specific methods can be found in the paper of Wickenden (2000). Below a summary of the most common approaches used in human pain research is given.

The first group of methods could be organized around intracellular recordings, where microelectrodes penetrate the cell membrane. Intracellular methods are used primarily *in vitro* and allow to study biophysical properties of individual cells involved in nociceptive signaling or properties of specific ion channels. The opportunity to gain human nociceptive cells via induced pluripotent stem cell technology and gaining those cells from organ donors or postsurgical tissue allows a detailed analysis of single neurons *via* electrophysiological methods (Namer et al. 2019, Zhang et al., 2019b).

The second group are extracellular recordings which can be obtained *in vitro* from human nerve biopsies (Quasthoff et al., 1995) and *in vivo* using the technique of microneurography (Vallbo, 2018; Ackerley and Watkins, 2018; Namer et al., 2009). Using pharmacological tools, excitability of the nerve fibers and other functional properties can be assessed. Nerve biopsies can be used to record the compound action potential of all C-fibers that the nerve contains. Thus, this method is inaccurate because nerve biopsy contains also non-nociceptive thermo- and mechano-receptors and not all C-fibers are nociceptive. In turn, microneurography allows recording signals from single nociceptors in awake humans. For that a thin needle microelectrode is inserted into a peripheral nerve and extracellular potentials of the single axons that are closest to the needle electrode can be recorded. Microneurography of C-nociceptors, due to factors such as low signal-to-noise ratio, is missing reliable spike sorting methods and therefore discharge patterns are challenging to obtain [see (Kutafina et al., 2022) for more details]. Thus, analyses of microneurographic recordings are often restricted to indirect assessment of nerve activity *via* latency changes of electrically induced test signals, called “marking method” (Schmelz et al., 1995). Rate of action potentials and frequency of action potentials in nociceptors have been found to correlate with pain intensity when stimuli like heat are used to activate those nerve fibers (Torebjörk, 1985). However, knowledge is limited to specific discharge patterns in nociceptors signaling physiological and pathological pain. Nociceptors not only conduct signals in the form of action potentials, but process the input. The frequency of action potentials and excitability is modulated by previous activity of nociceptors (Weidner et al., 2002; Bostock et al., 2003). Spontaneous activity in sleeping nociceptors has been found to correlate to ongoing neuropathic pain and is currently regarded as an objective biomarker for ongoing neuropathic pain.

One of the most widely used and methodologically well-established techniques of non-invasive electrophysiology is electroencephalography (EEG). For EEG, a highly variable number of electrodes (from 3 to 512 and more) can be used to move on the trade-off scale between mobility, price, and comfort to signal quality, spatial resolution and 3D source reconstruction reliability. EEG is capturing electrical activity of the brain (cortex primarily) and its large networks. This includes not only collective electrical signals from multiple actions potentials, but also, for example, synaptic and subthreshold (not sufficient to cross action potential threshold) activity (Buzsáki et al., 2012). The analysis of the EEG signal is primary based on spectral characteristics, as opposed to cell-level methods, in which the analysis focuses more on binary coding of neural firing. Examples of EEG research on pain perception include identification of brain activity during noxious stimulation (Tayeb et al., 2020) and, more generally, perspectives of EEG as a biomarker and measurement tool for pain (Zis et al., 2022; Zhang et al., 2012). Importantly, recent works suggest that it is important to shift the attention from the classical local spectral analysis towards brain networks and functional connectivity (Ta Dinh et al., 2019; Modares-Haghighi et al., 2021).

The non-invasive method of EEG is complemented by electrocorticography (ECoG), also known as intracranial electroencephalography (iEEG). ECoG electrodes are placed subdural (i.e., directly on the brain surface), providing a high-quality signal, but the invasiveness of the method limits the usage in humans to medically necessary cases such as pre-surgical monitoring in epilepsy (Jayakar et al., 2016). However, in animal studies ECoG can be used, for instance, to monitor the effects of anesthesia on mice (Schmidt et al., 2021).

Assessing local field potentials (LFP) (Kajikawa and Schroeder 2011) is another electrophysiological approach, which can be used *in vivo* and *in vitro*. The nature of LFP remains a point of discussion (Buzsáki et al., 2012). In contrast to surface electrodes as used in ECoG, LFPs are recorded from arrays of microelectrodes in the extracellular brain space. As ECoG, LFP is limited to *in vivo* usage in medically justified cases in humans. For instance; Huang et al. (2016) used LFP post-surgically to study deep brain stimulation (DBS) efficiency in patients with neuropathy. In animal models LFP has a much wider range of possibilities including recording the data in freely behaving animal subjects under different pain-related conditions (Song et al., 2021; Fu et al., 2018).

#### 3.3.2 Brain imaging

Enormous technical advancements in the last years have improved the possibilities of functional human brain imaging massively, which has resulted in a widespread and very common use in human pain research (Davis, 2019; Tracey, 2021). In particular, major progress in magnetic resonance imaging (MRI) not only in terms of technical capabilities related to data acquisition, but also with respect to availability and usability of analysis tools, has made MRI a common assessment tool (Logothetis, 2008). Functional MRI (fMRI) allows to assess regional changes in blood oxygenation levels in the brain (i.e., blood-oxygen-level-dependent, BOLD, imaging), for example, in response to external stimuli such as nociceptive stimulation or tasks participants perform, but also in resting states, with no active task performance or stimulation (“mind-wandering”). A huge variability in different experimental designs and/or specific imaging sequences, resulting for example in different spatial or temporal resolution, can be implemented to answer different research questions on brain responses on the perception of experimental, acute, or chronic pain [e.g., (Martucci and Mackey 2016; Vachon-Presseau et al., 2016)].

Currently, the most common approach when using fMRI is to assess activations (and/or deactivations) across the whole brain or within specific smaller regions of interest (*a priori* defined, based on previous results and by using e.g., anatomical atlases). Following such a straightforward approach, a seminal study described already in 1999, a set of distinct brain regions that are activated in a stimulus intensity dependent manner in response to experimental pain (Coghill et al., 1999). This specific set of brain regions activated in response to experimental pain has been confirmed in many later papers [(Apkarian et al., 2005; Schweinhardt and Bushnell 2010), for review]. With that, these fMRI studies also convincingly confirmed that the perception of pain associated with activation in a distributed brain network rather than in one specific brain region (Apkarian et al., 2005; Schweinhardt and Bushnell, 2010). It is important to point out that the activation of this brain network is not specific to pain. All regions of this network are also implicated in the processing of many other stimuli (e.g., other sensory modalities, emotion, cognition). The same applies to the network as a whole (Mouraux et al., 2011; Iannetti and Mouraux, 2010). For this reason, it is not possible to describe a distinct system or network of brain regions related to the processing of pain in humans. While there is a set of some few structures typically involved in the processing of pain (e.g., insula, anterior cingulate cortex, thalamus) (Apkarian et al., 2005; Schweinhardt and Bushnell 2010), the exact processing brain circuit can largely vary depending on the functional relevance of the pain (e.g., acute vs. chronic pain, static pain sensitivity vs. dynamic pain modulation). In addition to assessing brain activation in response to a stimulus, functional connectivity could be described. Simply speaking, functional connectivity describes how different brain regions show a positive or negative coupling of their activity time series (Park and Friston, 2013). This approach allows describing brain networks in terms of their coupled activity. It is assumed that this type of temporal coupling in brain responses indicates common underlying processing. Recent data confirms and emphasizes the importance of such functional networks. For example, it has been proposed that alterations in functional connectivity, specifically between the nucleus accumbens and the ventromedial prefrontal cortex, predict whether people with subacute back pain develop chronic pain (Baliki et al., 2012; Löffler et al., 2022). It is worth mentioning that functional connectivity is a type of analysis that is not restricted to fMRI data. It can be also used, for example, with EEG data, broadening the range of potential insight, in particular if fMRI and EEG measures are combined (Ebrahimzadeh et al., 2022).

Generally, fMRI can be used to assess brain responses during clinical pain episodes as well as to experimentally induced stimuli. Both approaches are important and needed, because naturally brain responses to clinical and experimental pain differ. Nevertheless, reliable and valid assessment of brain responses, e.g., during a clinical pain attack, is from a methodological point of view more challenging, because clear and usable time points/trigger for the analysis are missing. MRI is a major and often used technique in the context of human brain imaging in pain research, but there are also other related techniques. One example is functional near-infrared spectroscopy (fNIRS) that allows, similarly to fMRI, to assess hemodynamic activity in the brain (Hu et al., 2021; Karunakaran et al., 2021). While fNIRS has the major advantage that it is portable and thus can be also used at the bedside, its measures are restricted to brain regions near the cortical surface. Pain is known to activate several sub-cortical brain regions such as the thalamus and parts of the basal ganglia as well as “hidden” structures such as the insula and anterior cingulate cortex, rendering the use of fNIRS challenging. Despite these considerations, fNIRS can be valuable particularly in assessing vulnerable populations for which fMRI assessments are impossible or very stressful.

Despite the advancements of recent years in these brain imaging techniques, there are still several limitations and open questions. While (f)MRI offers a much better spatial resolution compared to EEG and similar techniques, the temporal resolution is still restricted. This is not only because of technical limitations—actually, new imaging sequences have improved the possible temporal resolution strongly without affecting spatial resolution—rather, the BOLD response itself is a much slower signal than the underlying electrical neural pulses. Nevertheless, a convincing coupling between such electrical pulses, indicating brain signaling, and the BOLD response has been shown (Logothetis, 2003; Logothetis, 2008). Even more important, brain imaging cannot be used as an objective biomarker of perceived pain in individuals. Although there have been efforts to establish such MRI biomarkers (Tracey 2021; Zhang et al., 2021), all these attempts failed so far to predict perceived subjective pain reliably and on an individual level (Davis et al., 2020; Davis et al., 2012). While newer sophisticated statistical methods, using multivariate techniques and/or machine learning approaches, have been shown to successfully differentiate, for example, physical and emotional pain based on the brain signatures (Wager et al., 2013; Woo et al., 2015), these methods cannot be easily generalized to more dynamic pain modulation and to chronic pain (López-Solà et al., 2017; Woo et al., 2017; Zunhammer et al., 2018). Moreover, these predictions cannot be applied (yet) on a single subject level to predict individual pain perception. Correspondingly, there is an intense discussion ongoing whether this level can be reached at one point and what with would mean in ethical but also legal context (Davis et al., 2012; Davis et al., 2020; Mackey et al., 2019).

### 3.4 Assessments of output measures

Pain is defined as a subjective experience which, accordingly, cannot be measured objectively. Nevertheless, there are possibilities to use reactions of the autonomic nervous system as correlates of the processed nociceptive input as well as behavioral indicators and self-reports including rating scales and questionnaires to gain estimates of the pain a person experiences.

#### 3.4.1 Physiological assessments

Most physiological manifestations discussed below are not specific for pain, but rather reflect the balance between the sympathetic and parasympathetic systems. Thus, such physiological manifestations can be used as indirect read-out of the distress pain causes. Nevertheless, in a given measurement context such read-outs can provide useful information. For example, in the recent decades the development of high-quality wearable sensors allows to collect out-of-the-lab long-term data and simple physiological markers have large potential to contribute to the improvement of the individual patient’s wellbeing through personalization of the interventions. Physiological measures could serve as support in assessment of unconscious patients, or patients with limited communication options. Arguably, the three modalities with the most potential for out-of-the-lab use are: breathing patterns, heart rate and skin impedance.

##### 3.4.1.1 Breathing pattern (BP)

The review of Jafari and colleagues (Jafari et al., 2017) provides a summary on the bi-directional links between respiration and pain. Different parameters of respiration are considered with the simplest parameter to measure in practice being the breathing rate.

Respiration patterns are tightly connected to cardiac activity. The synchronization of breathing rate and heart rate, and the potential of controlling those parameters through dedicated exercises is a field of extensive research (Schäfer et al., 1999; Perry et al., 2019). In a more specific context of pain and nociception, the combination of signals based on ECG, respiration and BP is used in the study of Devalle and colleagues (Devalle et al., 2018) to differentiate responses to nociceptive stimulation in minimally conscious state (MCS) and unresponsive wakefulness syndrome (UWS).

##### 3.4.1.2 Heart rate variability (HRV)

Heart Rate Variability (HRV) has been gaining attention in recent decades (Rajendra Acharya et al., 2006). The advantage of HRV is its strong correlation to the sympathetic-parasympathetic balance coupled with data collection simplicity: unlike the complete ECG signal, the large R peaks needed for HRV, can be extracted even from comparatively noisy recordings. Kasaeyan Naeini et al. (2021) used machine learning methods applied to HRV data to differentiate pain levels in postoperative patients. Forte et al. (2022) recently published a systematic review of HRV in pain research and concluded that HRV should be considered as a promising index and tested further.

##### 3.4.1.3 Skin impedance (galvanic skin response, GSR or electrodermal activity, EDA)

Changes in skin impedance is another non-specific measure. Hampf (1990) showed that skin impedance changes are more prominent than the heart rate increase during the cold pain stimulation (no HRV markers were considered). In the paper of Ghita et al. (2020) the authors show that it can be an efficient indication for acute pain experience, at least in a lab setting with a possibility of a baseline measurement.

##### 3.4.1.4 Cortisol levels

Cortisol is known as an important stress biomarker [see (Hellhammer et al., 2009) for the discussion of benefits and potential weaknesses]. Measuring cortisol levels with wearables, such as described in (Parlak et al., 2018), could carry a potential for monitoring of chronic pain, as hormonal abnormalities have been discussed as pain biomarkers (Tennant 2013).

##### 3.4.1.5 Other methods

The surgical pleth index (SPI) (Ledowski et al., 2019) is a score based on pulse patterns used for anesthesia guidance. SPI is used intraoperatively for steering analgesia. It uses pulse-wave amplitude and the heartbeat interval. It represents a combined assessment of peripheral (sympathetically mediated) vasoconstriction and cardiac autonomic tone. Since it is recommended to be used only in patients during narcosis, it is a measure of vegetative nervous response to nociceptive input and not of subjective pain sensation. Another measurement used on anesthetized patients (Wildemeersch et al., 2018) and in sedated critically ill patients (Vinclair et al., 2019) is pupil’s reflex papillary dilatation.

#### 3.4.2 Self-reports and behavioral assessments

As mentioned above, the outcome measures described so far lack specificity for pain processing. While these measures can be still used as valuable indicators of processes related to the perception of pain (e.g., autonomic arousal due to painful experiences), we cannot use these measures as specific and sensitive assessments of the subjective experience of pain. Although it might sound surprising or frustrating, the only available reliable and valid methods to assess how pain is perceived are self-reports. While self-reports offer an invaluable window into the subjectivity of pain, this subjectivity in turn poses some major challenges to the assessment and measurement of pain, because no objective anchor can be used. In addition, self-reports themselves are affected by several cognitive and social factors. Typically, response biases and perceptual representations cannot be separated, albeit some computational models allow to separate these representations for specific behavioral assessments (see below).

##### 3.4.2.1 Rating scales

Rating scales are an omnipresent assessment method of perceived pain in experimental and clinical contexts (Dworkin et al., 2005). As a fast and easy to use method such ratings scales fulfil essential functions. Rating scales for pain can come in many different types and shapes (Williamson and Hoggart, 2005). Most common are categorial, numeric and visual analogue scales. While categorial scales offer a certain number of categories as descriptors, for example of the intensity of pain, numeric scales provide a range of numbers to rate pain intensity or other aspects of pain. Common numeric scales range from 0–10 or from 0–100. Visual analogues scales in contrast, provide only a horizontal or vertical line with only very few anchors, typically only the end anchors. The rating is given on such scales by marking the location on this line corresponding to the own perception and relative to the anchors. In addition, other pictorial scales are sometimes used, for example, in children, using faces/smileys. While categorial and pictorial scales are easy to understand and thus can be also used reliably in children and people with cognitive deficits, they are less precise and detailed (Kremer et al., 1981; Williamson and Hoggart, 2005). Nevertheless, while numerical and visual analogue scales offer more detail, the resulting data has to be viewed and analyzed with caution. Although the ratings are numerical, these numbers cannot be treated on an interval scale, but only at the level of an ordinal scale (Schweiker et al., 2017).

Most commonly, rating scales are used to assess the perceived intensity of pain. In addition, particularly in experimental settings, the perceived unpleasantness of pain is assessed as another and different aspect of the pain experience (Price 2000; Rainville et al., 1997). Other aspects of the pain experience can be assessed as well, for example, suffering (Loeser and Melzack 1999; Bustan et al., 2015; Löffler et al., 2018), but these ratings often show a high overlap and multicollinearity because these aspects of pain are not perceived as distinct entities (Becker et al., 2020a; Becker et al., 2020b). Pain is a multidimensional experience for which the different dimensions typically go hand in hand.

Importantly, it has been shown that the specific end anchors of rating affect the ratings. One of the most common end anchors is “most intense pain imaginable.” However, this anchor has been criticized for not been very vague and with huge interindividual differences (Dannecker et al., 2007). Specifically, patients suffering from chronic pain appear not to be able to identify well with this anchor (de Williams et al., 2000; Yokobe et al., 2014). Moreover, evaluations on ratings scales are strongly affected by other cognitive and social factors. For example, social desirability affects pain ratings of experimental as well as clinical pain (Deshields et al., 1995; Haythornthwaite et al., 1991; Kermit et al., 2000; Komarahadi et al., 2004). Further, individuals have different understandings of the descriptors and anchors of pain, which can lead to a difference in usage of the scales and thus the ratings of pain (Becker et al., 2020a; Becker et al., 2020b). Despite these influences, rating scales are an invaluable tool as an outcome measure, but one has to be aware of the effects of such modulatory factors (Dworkin et al., 2005). In particular, such factors have to be considered in computational models of pain to gain a comprehensive and valid estimation of pain processing in humans (see Section 3.4.3 below).

##### 3.4.2.2 Questionnaires

Clinical pain as well as pain-related cognition, emotions, and personality states and traits are often assessed with questionnaires. A large array of validated questionnaires assessing various aspects related to the perception of pain is available. In contrast to rating scales, such questionnaires typically do not assess the current experience of pain but rather characteristics of ongoing pain such as its temporal pattern and functional impairments by the ongoing pain. Reviewing all available pain-related questionnaires is beyond the scope of this review. For illustration only a few commonly used examples are mentioned. As such, the West Haven-Yale Multidimensional Pain Inventory (Kerns et al., 1985) focuses on impairments by pain in addition to perceived support by significant others. The McGill Pain Questionnaire (Melzack 1975), in contrast, focuses on a fine-grained description of the perception of present pain using many adjectives, such as burning, throbbing, flashing, etc., with the aim to describe sensory, affective, and evaluative components of pain. While the McGill questionnaire is often used in clinical contexts, it can be also used to assess the perception of acute and experimental pain.

Other commonly used questionnaires focus more on the cognitive and emotional aspects of evaluating pain. For example, the Pain Catastrophizing Scale [PCS; (Sullivan et al., 1995)] assesses the tendency to catastrophize when confronted with pain and the Fear of Pain Questionnaire [FPQ-III; (McNeil and Rainwater 1998)] assesses specifically how fearful a person is considering pain in specific situations. Similarly, the Fear-Avoidance Beliefs Questionnaire [FABQ; (Waddell et al., 1993)] assesses patients’ belief on how physical activity and work affect pain, specifically back pain.

##### 3.4.2.3 Behavioral assessments

The perception of pain typically results in behavioral output such as escape or avoidance behavior, protecting or relieving postures, mimicking etc. In turn, such output can be assessed as indicators of perceived pain. However, as with several other measures, such behaviors are not necessarily specific to pain. One exception is mimicking or grimacing. It is known that the perception of pain results in specific changes in human facial action units, i.e., specific activity of facial muscles or groups of muscles (Kunz et al., 2019; Hassan et al., 2021). The Facial Action Coding System [FACS; (Ekman et al., 2002)] provides a taxonomy of facial movements as physical expressions of emotions in general and specifically including pain. Based on this system, the facial expression of pain can be used as an indicator of perceived pain based on which this system is particularly useful in non-verbal populations, for example, people with (severe) dementia (Kunz et al., 2021; Lautenbacher et al., 2018). Because of the specificity using mimic is an invaluable tool to assess pain, although it only gives very rough estimates of the magnitude of the perceived pain. In addition, a substantial proportion of people, in particular adults, show none or only weak facial responses to pain (Kunz et al., 2019). In experimental settings, an often-used behavioral outcome is reaction time, for example, in tasks in which fast enough responses (e.g., button presses) enable the avoidance of a painful stimulus [e.g., (Roy et al., 2014; Gandhi et al., 2022)]. Such tasks allow assessing, for example, the motivation to avoid or escape pain. The relevance of the behavioral outcome measure is defined by the task, i.e., if the behavioral response is directly related to pain processing, because the behavior is again not specific to the perception of pain. Interestingly, behavioral outcomes such as reactions times in forced-choice decision-making can be used in computational models to differentiate components of perceptual processes [e.g., (Wiech et al., 2014; Becker et al., 2020a; Becker et al., 2020b)]. Such models can provide mechanistic insights and are important tools to increase our understanding of human pain perception. As such, these computational models have been gaining more and more attention recently in pain research.

#### 3.4.3 Modulatory factors of measuring output

The perception of pain is strongly modulated by factors other than the characteristic of the external stimuli. The number of such potential factors is very high, and a comprehensive review is beyond the scope of this paper. To highlight the importance of such modulatory factors, we describe a few examples. Emotional-motivational and cognitive factors are known to strongly affect how experimental and clinical pain is perceived (Bushnell et al., 2013). For example, the same nociceptive input is perceived as more intense and more unpleasant when being in a bad mood, while it is perceived as less intense and less unpleasant in a good mood (Rhudy et al., 2007). Effects of such an emotional modulation have been shown not only on self-reported pain, but also on the RIII reflex as an indicator of spinal nociceptive processing (Rhudy et al., 2005). Similarly, attention to and distraction from pain has a strong modulatory effect, with distraction having pain-inhibiting and attention having pain-facilitatory effects (Dunckley et al., 2007). Importantly, such effects of attention and distraction have been confirmed in animal studies, confirming a spinal involvement of such top-down effects (Bushnell et al., 1985). The modulatory effects of emotion and distraction have been demonstrated to depend on different brain circuits (Villemure and Bushnell 2009), highlighting that the modulation of pain perception can be exerted through different mechanisms.

Apart from such psychological factors, biological and environmental factors have been demonstrated to modulate the perception of pain as well. For example, the hormonal status in women is related to variation in pain perception as well as age (Vincent and Tracey 2008). Furthermore, temperature and humidity of the environment can affect the pain perception (Fagerlund et al., 2019; Li et al., 2019) as examples of environmental factors.

In addition to active experimental manipulation of such modulatory factors, potential influences can be assessed by using the publicly available data (weather, air pollution, traffic intensity etc.), personal smart sensors (barometric pressure, air humidity), and self-report tools such as electronic diaries (coffee intake, social interactions, physical activity).

## 4 Discussion

The aim of the presented overview is to facilitate the development of interdisciplinary projects on pain research through outlining the landscape of available methods for assessments of pain perception, indicators of nociception, and relevant neural structures. One of the most important points we wanted to bring to the reader’s attention is the difference between the experience of pain and any measurable physical manifestations. While pain and nociception are of course closely linked, those links can be more complex and the target of many modulatory influences (Mouraux and Iannetti 2018). To avoid confusion, it is essential to use appropriate terminology and clearly distinguish between pain, nociception, and linked physiological manifestations.

On the side of the “input” several aspects have to be considered when investigating pain and/or nociception. The right modality has to be picked, activating the right proportion of peripheral nociceptors, and the right application mode, e.g., threshold versus suprathreshold testing, phasic or tonic stimulation has to be chosen, to give specific information about certain pathways or aspects of pain perception. The mechanisms underlying chronic pain are different from the perception of acute and/or evoked pain. Acute pain serves the purpose of warning an individual about (potential) tissue damage, while chronic pain has lost this purpose. Even within acute pain, different mechanisms might be involved. For example, warning functions for acute pain coming from outside such as a hot object possibly causing burning relies on a different mechanism compared to the situation when something has already entered the body and changed the inner homoeostasis such as inflammation. It is not only important to consider which nociceptive pathways are targeted by specific tests, but also to which clinically relevant pain state these pathways and their study might contribute. Considering such different levels and their potential interaction adds complexity to pain assessments. One example of such complexity is measuring heat pain threshold in patients with ongoing neuropathic pain, although this sounds like an easy task. The subclass of mechanosensitive C-fibers together with A-delta fibers determine the heat pain threshold in humans. However, a major contributor to the peripheral component of ongoing neuropathic pain is spontaneous activity in another subclass of nociceptors, namely the sleeping nociceptors, thus, complicating this easy task in terms of underlying and contributing mechanisms. In addition, while threshold assessments might indicate pain sensitivity, such threshold assessments are most likely not well targeted to investigate mechanisms responsible for ongoing pain. On the side of the “output”, it has to be kept in mind that measures that assess the perceived pain experience based on self-reports are first of all subjective and cannot be validated based on any objective measures or linearly related to input. Second, self-reports are easily confounded by many internal and external factors that are unrelated to the core pain experience, such as the current bodily state, response bias, social desirability, expectations, individual learning history etc. Other output measures assess various physical and behavioral functions, which might be important indicators, for example, of reactions of the vegetative nervous system. Nevertheless, all these assessments are not specific to the pain systems, meaning that they can be used to achieve a comprehensive picture of responses to pain, but they cannot be used as standalone indicators of perceived pain in humans. Moreover, it has to be taken into account that behavioral and vegetative responses can be altered in many ways in chronic pain, but such alterations do not necessarily indicate specific alterations due to the ongoing pain. For example, responses of the vegetative nervous system might be altered due to small fiber neuropathy as well as due to changes in the central nervous system or cognitive evaluations of the pain.

Taken together, the whole process of pain signaling starting spontaneously or as a result of external stimuli is extremely complex. However, this is exactly the point where network-type computational models that integrate different data modalities can help. As a comparatively simple example, on the level of brain responses recent advances in the field of fMRI emphasize the importance of functional connectivity with networks of brain regions, which appears to be more informative than activation in single brain regions. Increasing the level of complexity, integrating processes at different levels of nervous system, and adding both structural and functional assessments will result in novel and important mechanistic insights in human pain perception in acute and chronic states. Animal models foster this process because they can serve as surrogate models where data assessments in human data are limited or not possible (e.g., *in-vivo* invasive electrophysiology, genetic modifications). However, to build valid and reliable multimodal network models, interdisciplinary collaboration is needed at least between clinicians, psychologists, life scientists, and computational experts. Moreover, the most important reason to study (human) pain should be kept in mind as well, which is to help patients suffering from pain. For this reason, the outlined approaches and resulting models can be supported and improved also by the interactions with professionals such as nurses, social workers, clinical psychologists, and biomedical ethicists as well as patients themselves.

Finally, to conduct multidisciplinary collaborations and build transparent and reproducible models, best practice of data handling, such as FAIR (findable, accessible, interoperable, reusable) principles (Wilkinson et al., 2016) needs to be applied as well as the rules of Good Clinical Practice in general. Further, to allow sharing data, particularly clinical data, in order to test and improve developed modes, better legal and technical solutions, in particular data standardization and (inter-)national databases, need to be developed.

In sum, the importance of building multi-modal network models has been discussed in the context of emerging field of network physiology (Bashan et al., 2012; Ivanov PCh, 2021) but also in the context of precision medicine and digital twins (Coorey et al., 2021; Björnsson et al., 2019; Lonsdale et al., 2022). Some promising and valuable computational models have been already developed in the field of human pain [see e.g., (Lang et al., 2021; Ionescu et al., 2019)] and a review of such models could help to understand, predict and analyze nociceptive system function and pain perception. Accordingly, we are very optimistic that the domain of multiscale and network-oriented computational pain research will continue to grow and contribute to our understanding of human pain signaling and perception in health and disease states. This scientific direction and the underlying collaborative work have the potential to move pain research domain to a completely new level in finding new targets for pain relief and development of personalized medicine.
